# Exploring gonadotropin dosing effects on MII oocyte retrieval in ovarian stimulation

**DOI:** 10.1007/s10815-024-03102-z

**Published:** 2024-04-04

**Authors:** Krystian Zieliński, Anna Kloska, Piotr Wygocki, Marcin Zieleń, Michał Kunicki

**Affiliations:** 1 INVICTA Research and Development Center, Sopot, Poland; 2 Department of Biomedical Engineering, Faculty of Electronics, Telecommunications and Informatics, Gdańsk University of Technology, Gdańsk, Poland; 3 Department of Medical Biology and Genetics, Faculty of Biology, University of Gdańsk, Gdańsk, Poland; 4 MIM Solutions, Warsaw, Poland

**Keywords:** Ovarian stimulation, Gonadotropin dosage, IVF, MII oocytes, IVF, Machine learning model

## Abstract

**Purpose:**

Ovarian stimulation with gonadotropins is crucial for obtaining mature oocytes for in vitro fertilization (IVF). Determining the optimal gonadotropin dosage is essential for maximizing its effectiveness. Our study aimed to develop a machine learning (ML) model to predict oocyte counts in IVF patients and retrospectively analyze whether higher gonadotropin doses improve ovarian stimulation outcomes.

**Methods:**

We analyzed the data from 9598 ovarian stimulations. An ML model was employed to predict the number of mature metaphase II (MII) oocytes based on clinical parameters. These predictions were compared with the actual counts of retrieved MII oocytes at different gonadotropin dosages.

**Results:**

The ML model provided precise predictions of MII counts, with the AMH and AFC being the most important, and the previous stimulation outcome and age, the less important features for the prediction. Our findings revealed that increasing gonadotropin dosage did not result in a higher number of retrieved MII oocytes. Specifically, for patients predicted to produce 4–8 MII oocytes, a decline in oocyte count was observed as gonadotropin dosage increased. Patients with low (1–3) and high (9–12) MII predictions achieved the best results when administered a daily dose of 225 IU; lower and higher doses proved to be less effective.

**Conclusions:**

Our study suggests that high gonadotropin doses do not enhance MII oocyte retrieval. Our ML model can offer clinicians a novel tool for the precise prediction of MII to guide gonadotropin dosing.

## Introduction

Controlled ovarian stimulation (COS) by exogenous gonadotropins stimulates the development of follicles to obtain mature oocytes with a preserved reproductive potential for in vitro fertilization (IVF). The stimulation protocol is preceded by desensitization of the hypothalamic-pituitary-ovarian axis with gonadotropin-releasing hormone (GnRH) analogs (either agonists or antagonists) to suppress the natural hormonal fluctuations of the menstrual cycle. This is followed by the administration of gonadotropins, including follicle-stimulating hormone (FSH) and luteinizing hormone (LH), to stimulate follicular growth and maturation in the ovary. In the final step, human chorionic gonadotropin (hCG) or GnRH agonist trigger is administered to induce the final oocyte maturation and subsequent retrieval.

The optimal number of mature oocytes retrieved after ovarian stimulation is generally considered to be between 5 and 15 to obtain the best results of the IVF procedure considered as live birth rate [1–5]. A poor response to stimulation may result in IVF cycle cancellation with no attempt to retrieve oocytes. However, the simple assumption that high doses of gonadotropins lead to better stimulation outcomes is misleading, as this increases the risk of ovarian hyperstimulation syndrome (OHSS) [4, 6–9]. Administration of gonadotropins at the lowest effective dose is, therefore, essential to achieve the desired result of stimulation while minimizing the side effects for the patient as well as the cost of treatment, recognizing the significant impact of medical expenses.

The choice of the most suitable gonadotropin dosing regimen is determined by factors influencing controlled ovarian stimulation outcomes, including age, body mass index (BMI), serum FSH, LH, estradiol, testosterone, prolactin, anti-Müllerian hormone (AMH), antral follicle count (AFC), and ovarian response to previous stimulations [10–18]. The varying correlation among predictors, such as AMH, AFC, and age, adds complexity to the gynecologists’ dosage selection owing to their cumulative influence, making comprehensive consideration almost impossible and dose selection challenging. The uncertainty of optimal gonadotropin dosages for effective ovarian stimulation raises concerns regarding overuse or suboptimal administration. To date, several studies have explored the impact of gonadotropin dosage size on IVF success outcomes, such as live birth rate, clinical pregnancy, good-quality embryos, or retrieved oocytes [3, 19–23]. However, in retrospective studies, gonadotropin dose alone can affect the outcome of stimulation, making it difficult to distinguish the respective roles of dose and other factors in the outcome.

The aim of our study was to develop a machine learning model to predict oocyte counts in patients undergoing IVF treatment and integrate these predictions with an examination of the impact of gonadotropin dosage to retrospectively ascertain whether a higher dose of gonadotropins translates into better ovarian stimulation results.

## Materials and methods

### Study population and dataset

This retrospective study utilized data from 9598 controlled ovarian stimulations conducted between November 2014 and July 2023. The data was collected from six INVICTA Fertility Clinics (Bydgoszcz, Gdańsk, Gdynia, Słupsk, Warszawa, Wrocław; Poland), with contributions from up to ten highly skilled gynecologists. The study adhered to the principles outlined in the Declaration of Helsinki and was approved by the Ethics Committee of the Regional Medical Chamber of Gdańsk (protocol code KB-23/20; approved on October 27, 2020). Written informed consent was obtained from all participants. Individual-level data were de-identified prior to the analysis.

Stimulations were conducted using menotropin (Menopur; Ferring GmbH, Kiel, Germany), follitropin delta (Rekovelle; Ferring GmbH, Kiel, Germany), or follitropin alfa (Gonal F; Merck Serono S.p.A., Modugno, Italy). Stimulations with other types of gonadotropins were excluded from the study.

The stimulation protocol (long or short, agonist, antagonist, or progesterone antagonist) and the initial gonadotropin dosage for the first cycle were routinely established by physicians based on the patient’s clinical profile, including parameters such as age, biomarkers of ovarian reserve (serum AMH level and AFC), BMI, infertility history, and previous (if not the first cycle) response to ovarian stimulation. Gonadotropin-stimulated patients were divided into three categories based on their potential response: high, normal, and poor. In the case of potentially high responders, short protocols with antagonists or progestins were applied flexibly. Gonadotropins were administered at doses of 150, 225, or 300 IU/day in either a fixed or flexible manner. In the second or subsequent stimulation cycles, dose adjustments were made after 3 days of stimulation to decrease the total dosage of gonadotropins (to avoid potential complications) and to decrease treatment costs. Dosage adjustments were made after the initial 3 days of stimulation based on ultrasound findings and/or estradiol levels or determined arbitrarily based on the physician’s experience and the patient’s response in the first cycle.

Stimulations with AMH levels below 0.01 ng/ml or above 15 ng/ml were excluded from the analysis due to their rare occurrence and the high variability of the model’s predictions. Additionally, for modeling purposes, stimulations with extreme MII/AFC values (> 20 MII oocytes retrieved or AFC > 50) were excluded to avoid bias in the analysis. For patients with a tendency to produce a high number of MII oocytes, a dedicated model and analysis should be performed. Furthermore, to minimize the impact of outliers, observations with an extremely low ratio of MII oocytes to AFC (< 20%) were also excluded. The features and parameters that describe the dataset used in this study, along with the summary statistics, are presented in Table 1.

**Table 1:** Summary statistics of the dataset

Parameter or feature	Mean ± SD (range) or cases (percentage)
No. of stimulations	9598
Age (years)	34.22 ± 4.42 (18–50)
BMI	23.52 ± 4.48 (14.70–64.47)
AMH level (ng/mL)	2.76 ± 2.14 (0.01–14.88)
AFC on the first day of stimulation	13.35 ± 8.05 (0–49)
MII oocyte count in the previous pick-up	6.30 ± 3.92 (0–25)
Glycemic abnormalities (diagnosed diabetes, hyperinsulinemia)	485 (5.05%)
Polycystic ovary syndrome	795 (8.28%)
Tubal factors infertility (tubal damage)	1248 (13.00%)
Ovulatory dysfunctions (premature ovarian failure, lack of ovulation)	750 (7.81%)
Uterine anomalies (dysmorphic, septate, bicorporeal, hemi-, aplastic uterus)	436 (4.54%)
Ovary abnormalities (ovarian cysts, absence of ova)	1414 (14.73%)
Uterus abnormalities (polyps, adhesions, myomas, endometrium disorder)	1770 (18.44%)
Endometriosis (endometriosis, endometrial cysts)	1636 (17.05%)
Idiopathic infertility	929 (9.68%)
Genetic defects (test results: abnormal karyotype, translocations, mutations, genetic defects)	4773 (49.73%)
Thyroid dysfunction (hypothyroidism, hyperthyroidism, thyroiditis, and abnormal TSH, fT3, fT4 lab values)	3192 (33.26%)
Other endocrine factors (DHEA deficiency^a^, testosterone deficiency, overactive adrenal glands, hypogonadism, growth hormone deficiency)	1072 (11.17%)
Male factor infertility (male factor, oligospermia, azoospermia, and semen test results^b^)	2454 (25.57%)
Reduced semen parameters (asthenozoospermia, teratozoospermia, decreased hyaluronic acid binding, elevated oxidative stress, abnormal postcoital test, abnormal semen parameters^c^	3065 (31.93%)
Stimulation protocol:	
Progesterone	1201 (12.51%)
Short antagonist	588 (6.13%)
Long antagonist	7157 (74.57%)
Short agonist	652 (6.79%)

*AMH* anti-Müllerian hormone, *AFC* antral follicle count, *BMI* body mass index, *DHEA* dehydroepiandrosterone, *fT3*, free triiodothyronine; *fT4*, free thyroxine; *SD*, standard deviation; *TSH*, thyroid-stimulating hormone

^a^DHEA levels below the lower limit range defined as 98.8–340 µg/dL (feature included because serum DHEA level positively correlates with AMH level in infertile women [24])

^b^Described as no sperm, sperm not found, no motile sperm, no live sperm, no sperm in the testicle, and no liquid sperm

^c^Non-reference values for ejaculate volume, pH, liquefaction time, liquefaction method, sperm count per ejaculate, sperm concentration per ml, proportion of progressively motile sperm, sperm DNA fragmentation, hyaluronic acid binding assay, viable sperm, normal morphology sperm, or dead sperm

### Prediction of the number of MII oocytes

A machine learning model [25] was utilized to predict the number of MII oocytes to be retrieved after ovarian stimulation using a dataset with the features described in Table 1. The gradient-boosting model [26] was trained using the LightGBM algorithm [27]. The gradients and Hessians of the loss function were calculated with respect to the predicted values and were used to update the model. The best-split point that maximized the reduction in the loss function was determined for each tree. Various regularization methods were used to prevent overfitting, including feature bagging, data bagging, and L1 and L2 regularization on weights. The maximum depth and maximum number of leaves in each tree were set to ensure weak learners. The hyperparameters of the model were determined using random grid hyperparameter optimization. This method randomly samples values from a predefined range of hyperparameters to find the best set that produces the highest cross-validation score. The performance of the model was evaluated based on the root mean square error (RMSE) score. The random grid search method can efficiently explore the hyperparameter space and find a suitable set of hyperparameters for the model without exhaustively searching the entire space. This is particularly useful for high-dimensional search spaces where an exhaustive search is computationally infeasible. The selected hyperparameters for the trained model are presented in **Listing 1**. Shapley additive explanation (SHAP) values [28] were used to explain the model.

Listing 1. Hyperparameters used for modeling in this study
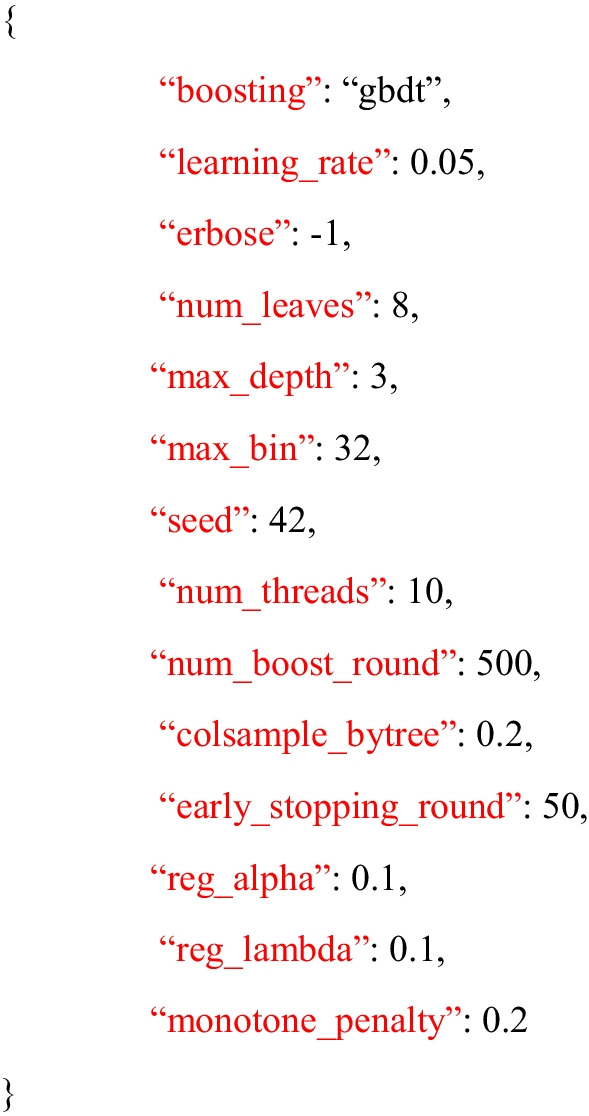


### Statistical analysis

The Shapiro–Wilk test was used to determine normality, and Levene’s test was used to assess the equality of variances. The x-sided *t*-test or Kruskal–Wallis *H* test was used to determine differences between the comparative groups. Dunn’s test was used for post hoc comparisons. Statistical significance was set at *P* < 0.05. All data were analyzed using statistical packages for Python 3.8.

## Results

### Model results

**Fig. 1:**
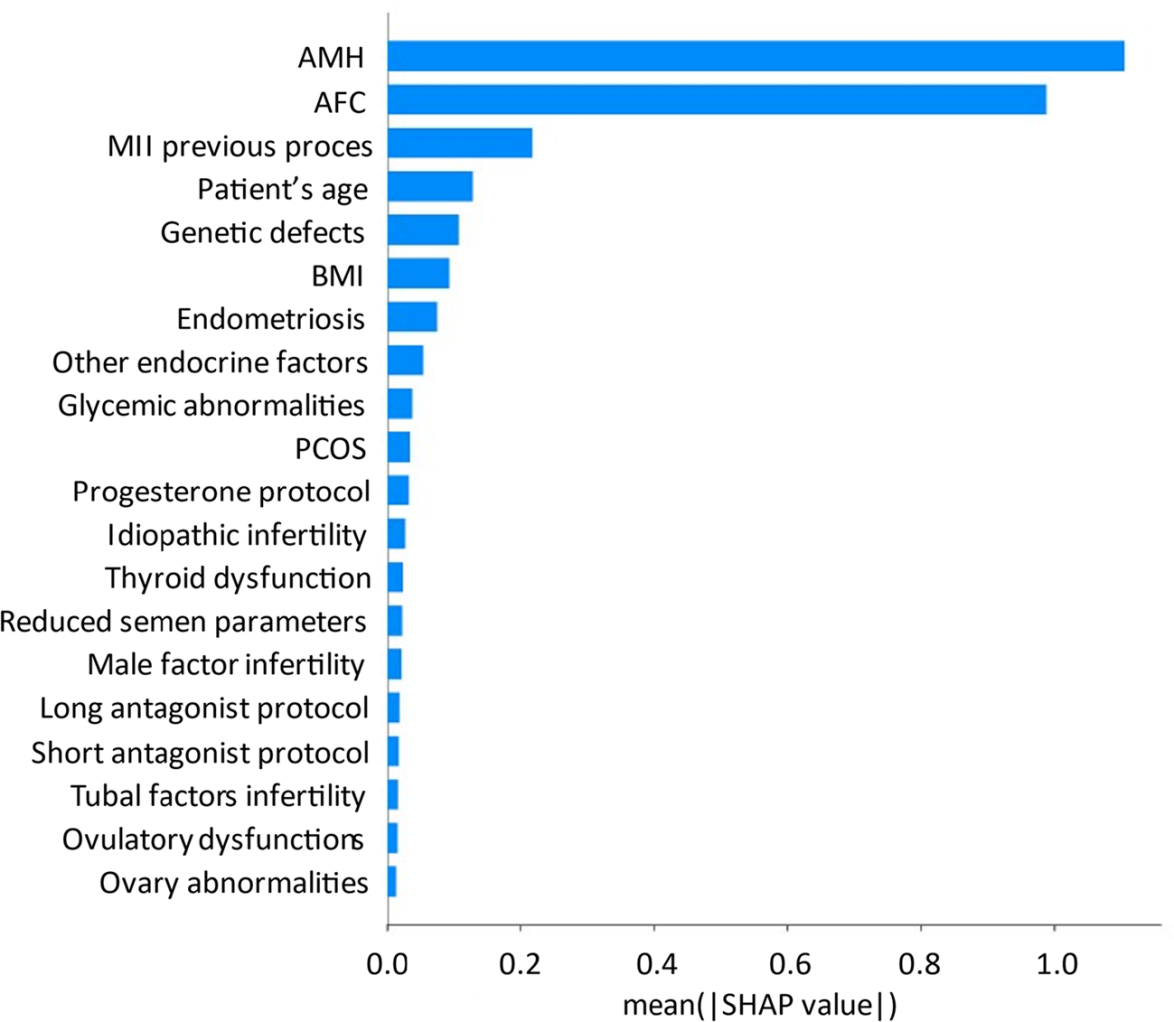
Ranking of the most important features for the prediction of MII oocyte number using the machine learning model. Features are sorted by their mean Shapley additive explanation (SHAP) values representing the average impact of a given feature on the model output magnitude. AMH, anti-Müllerian hormone; AFC, antral follicle count; BMI, body mass index; PCOS, polycystic ovary syndrome

Models were trained using fivefold cross-validation to ensure the stability of the results. The model achieved the following metric values: RMSE = 3.14, mean absolute error (MAE) = 2.47, mean absolute percentage error (MAPE) = 0.51, and median absolute error = 2.05. The impact of each feature on the model’s output was explained using the SHAP values (Fig. 1).

For MII oocyte prediction, AMH and AFC were the two most important features in modeling. Next, the number of MII oocytes obtained in the previous simulation and patient age was considered. Accompanying symptoms and BMI were ranked as less important for MII prediction but were used in calculations to include their cumulative effect on the prediction. The MII predictions made for each ovarian stimulation in our dataset were used in a subsequent analysis to compare the efficacy of gonadotropin dosing on the stimulation outcomes.

### The effect of gonadotropin dosing regimen on ovarian stimulation outcome in relation to prediction

In clinical practice, there is no uniform gonadotropin dosage scheme for ovarian stimulation, and the dosage is personalized based on patient characteristics. Achieving adequate follicular growth often involves sequential treatment. Initially, the daily dose is set and maintained for several days. Subsequently, adjustments are made by either decreasing or increasing the daily dose, which continues over the following days.

Based on gonadotropin dose, controlled ovarian stimulations were assigned to the following dosing regimen groups: 150/150, 150/225, 150/300, 225/150, 225/225, 225/300, 300/150, 300/225, and 300/300 IU/day (the first and second numbers refer to the doses of gonadotropins administered on days 1–3 and days 4–7 of stimulation, respectively). To assess the effect of the gonadotropin dosing regimen on ovarian stimulation outcomes, we compared the predicted number of MII oocytes (obtained for each stimulation from the dataset using the machine learning model) with the actual number of MII oocytes retrieved after stimulation. The results, referred to as the MII ratio, across the various gonadotropin dosing regimens are presented in Table 2.

**Table 2:** The effect of gonadotropin dosing regimens used for ovarian stimulation on the number of retrieved MII oocytes compared to the prediction

Dosing regimen ^a^	*N* ^b^	Retrieved MII Mean ± SD	Predicted MII Mean ± SD	MII ratio ^c^	*P* value ^d^
150/150	1990	9.15 ± 3.91	10.11 ± 1.59	0.97	** < 0.01**
150/225	63	7.10 ± 3.71	7.47 ± 3.18	0.98	0.79
225/150	1847	8.33 ± 3.79	8.24 ± 1.60	1.01	0.32
225/225	1876	7.52 ± 3.92	7.15 ± 1.92	1.02	0.07
300/225	2398	5.66 ± 3.45	4.98 ± 1.97	1.01	0.39
300/300	1364	4.20 ± 2.98	4.27 ± 1.79	0.98	0.40

*N*, number of stimulations; *SD*, standard deviation

^a^Described as gonadotropin doses (IU/day) administered on days 1–3 and days 4–7 of ovarian stimulation, respectively

^b^Dosing regimens with *N* < 30 were excluded from the analysis

^c^Calculated as the number of retrieved MII oocytes per predicted MII oocytes

^d^*t*-test was used to verify dosing regimen efficacy, with significance considered at *P* < 0.05

We found that the gonadotropin dosing regimen of 150/150 IU/day resulted in significantly lower counts of retrieved MII oocytes than predicted. The dosing regimens employing gonadotropins at doses of 225 or 300 IU/day did not achieve statistical significance.

As these results suggest that some dosing regimens may have different effects on ovarian stimulation, we decided to perform a more detailed analysis by separately assessing the impact of FSH doses administered on days 1–3 of stimulation (starting dose) and days 4–7 of stimulation (continuation dose) on the stimulation outcome.

### The effect of starting gonadotropin doses on ovarian stimulation outcome

To determine whether increased starting gonadotropin doses led to a higher number of retrieved MII oocytes, we compared this parameter across different MII prediction groups and FSH doses administered on days 1–3 of ovarian stimulation. According to the Bologna criteria, poor ovarian response is defined as the retrieval of less than four oocytes [2], and a range of 5–15 oocytes retrieved after ovarian stimulation is considered to be optimal to ensure the success of the IVF process [1, 2]. Therefore, we decided to conduct our analysis in MII prediction groups distinguishing patients with suboptimal prediction of 1–3 oocytes and predictions in the optimal range of 4–8 and 9–12 oocytes. To compare the stimulation outcomes, we used the MII ratio, defined as the number of retrieved MII oocytes divided by the predicted MII oocyte count. This approach ensures that our conclusions are not biased by the more frequent assignment of high doses to patients with poor prognosis.

**Fig. 2:**
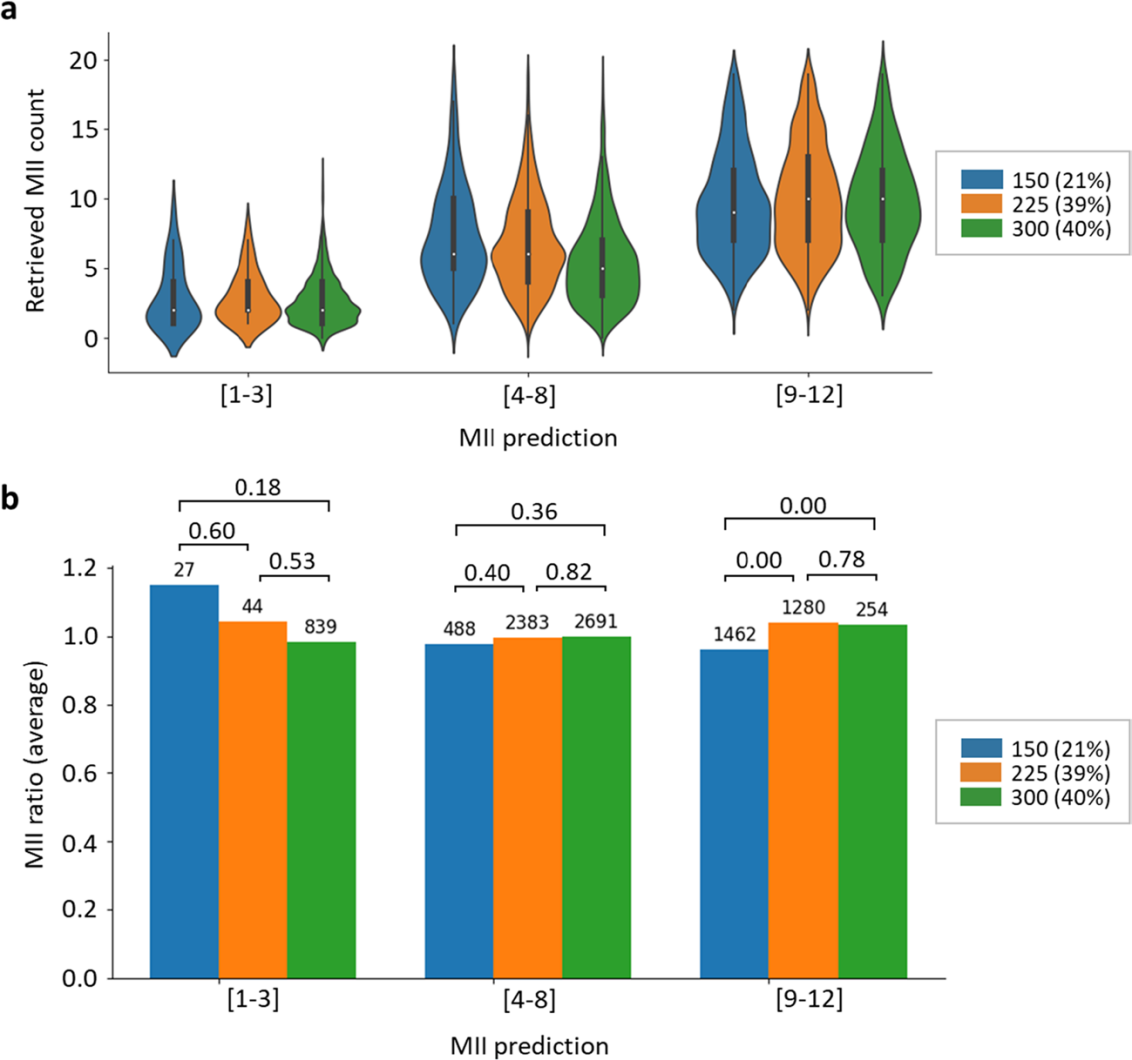
Effect of starting dose of gonadotropin on ovarian stimulation outcome. Comparison of retrieved MII oocyte counts to predictions across gonadotropin doses of 150, 225, or 300 IU/day administered on days 1–3 of ovarian stimulation. **a** Distribution of retrieved MII oocyte counts in various MII prediction groups at different gonadotropin doses. The graph displays the median (white dot), interquartile range (bold black line), minimum and maximum values (thin black line), and frequency (width of density plot). **b** Average MII ratio (defined as the retrieved MII/predicted MII) in various MII prediction groups across gonadotropin doses. The percentages of cases are indicated in parentheses in the legend. The numbers above the bars indicate sample sizes and *P* values for group comparisons (*t* test)

We found that increasing the dose of gonadotropins administered on days 1–3 did not significantly affect the MII ratio in patients with low (1–3) and intermediate (4–8) oocyte predictions (Fig. 2). However, in the group predicted to produce 9–12 oocytes, the MII ratio significantly increased in patients receiving 225 and 300 IU/day of gonadotropins in this timeframe compared with 150 IU/day.

These results suggest that higher starting doses of gonadotropins are not associated with increased oocyte retrieval in patients with low and intermediate MII predictions.

### The effect of continuation gonadotropin doses on ovarian stimulation outcome

To determine whether increased continuation doses of gonadotropins led to a higher number of retrieved MII oocytes, we compared the MII ratios across various MII prediction groups and gonadotropin doses administered on days 4–7 of ovarian stimulation.

**Fig. 3:**
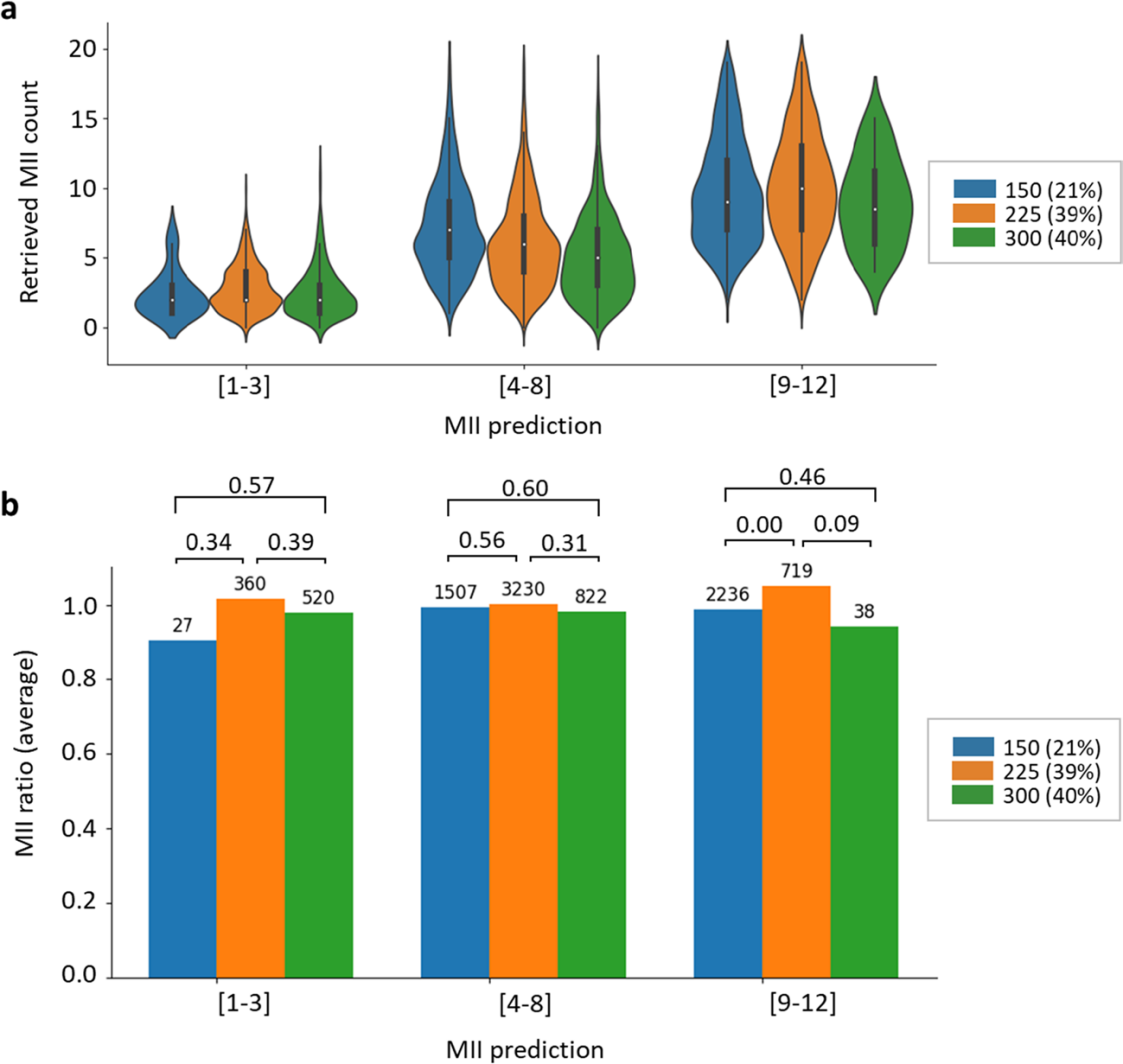
Effect of the continuation dose of gonadotropin on ovarian stimulation outcomes. Comparison of retrieved MII oocyte counts to predictions across gonadotropin doses of 150, 225, or 300 IU/day administered on days 4–7 of ovarian stimulation. **a** Distribution of retrieved MII oocyte counts in various MII prediction groups at different gonadotropin doses. The graph displays the median (white dot), interquartile range (bold black line), minimum and maximum values (thin black line), and frequency (width of the density plot). **b** The average MII ratio (defined as retrieved MII/predicted MII) in various MII prediction groups across gonadotropin doses. The percentages of cases are indicated in parentheses in the legend. The numbers above the bars indicate sample sizes and *P* values for group comparisons (*t* test)

We observed that increasing the gonadotropin dose administered during this period did not significantly change the MII ratio in patients with low (1–3) and intermediate (4–8) oocyte predictions (Fig. 3). Only patients predicted to have 9–12 oocytes responded significantly better to 225 IU/day than to 150 IU/day gonadotropins. However, the highest dose did not improve the outcome.

These results suggest that higher continuation doses of gonadotropins are not associated with increased oocyte retrieval in patients with low and intermediate MII predictions.

### The effect of cumulative gonadotropin doses on ovarian stimulation outcome

Similarly, we analyzed the impact of cumulative gonadotropin doses administered over 7 days on the stimulation outcome, defined as the MII ratio.

**Fig. 4:**
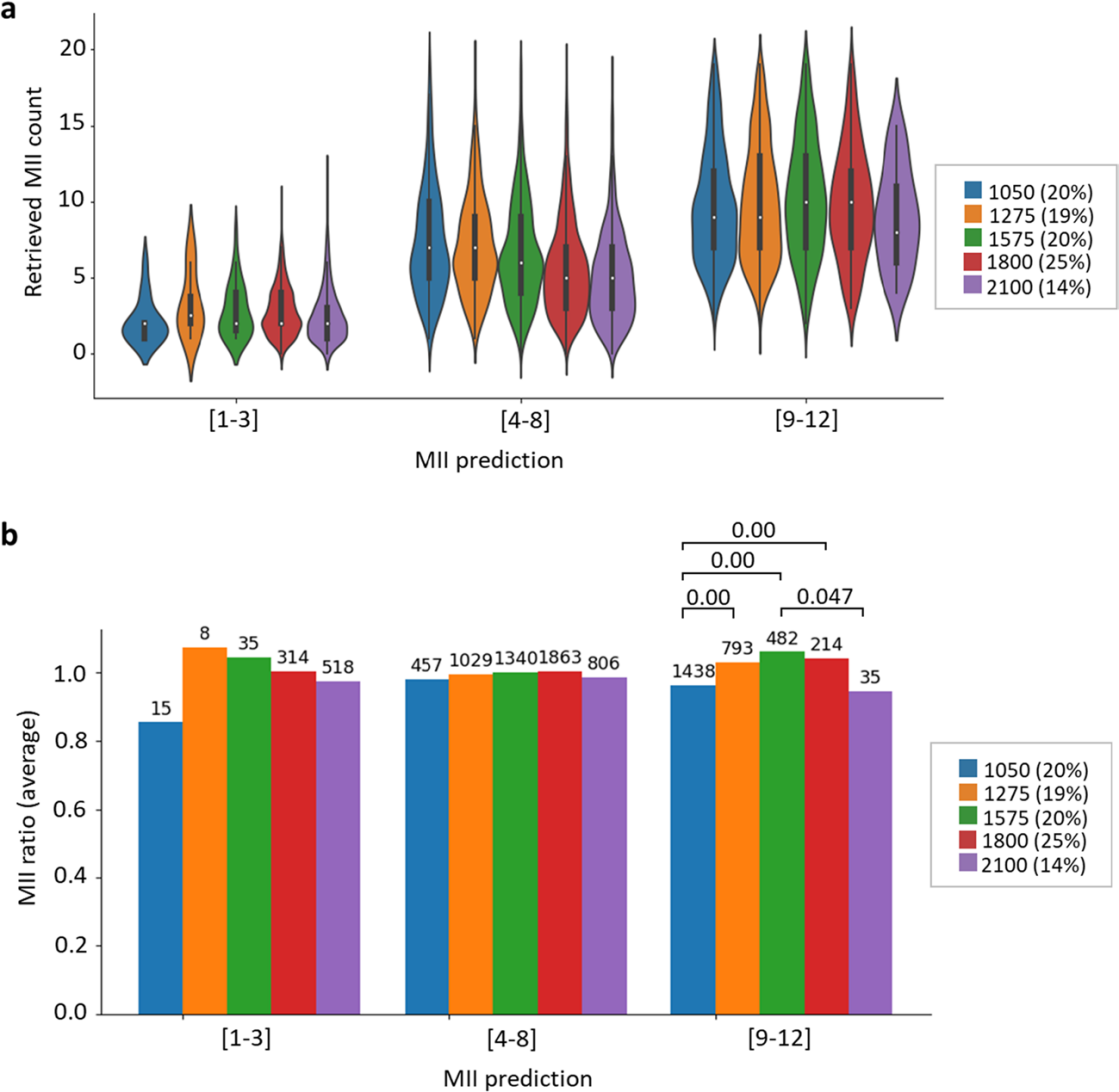
Effect of cumulative gonadotropin dose on ovarian stimulation outcomes. Comparison of retrieved MII oocyte counts to predictions across cumulative gonadotropin doses (IU) administered over 7 days of ovarian stimulation. **a** Distribution of retrieved MII oocyte counts in various MII prediction groups at different gonadotropin doses. The graph displays the median (white dot), interquartile range (bold black line), minimum and maximum values (thin black line), and frequency (width of the density plot). **b** The average MII ratio (defined as retrieved MII/predicted MII) in various MII prediction groups across gonadotropin doses. The percentages of cases are indicated in parentheses in the legend. The numbers above the bars indicate sample sizes and *P* values for group comparisons (Dunn’s post hoc test after the Kruskal–Wallis *H* test)

We observed that increasing the cumulative gonadotropin dose did not significantly change the stimulation outcome in patients with low (1–3) and intermediate (4–8) oocyte predictions (Fig. 4). In the intermediate prediction group, the MII ratio was similar regardless of the dose. In the MII prediction group (9–12 MII oocytes), both the lowest and highest cumulative doses were associated with a significantly lower MII ratio, whereas the most favorable impact was observed with cumulative gonadotropin doses spanning 1275 to 1800 IU administered over 7 days.

These results suggest that higher cumulative gonadotropin doses are not associated with increased oocyte retrieval in patients with low and intermediate MII predictions.

## Discussion

Our findings showed that increasing the dose of gonadotropins administered during ovarian stimulation for IVF was not associated with improved efficacy in terms of the number of retrieved MII oocytes, particularly among patients predicted to produce low (1–3) or intermediate (4–8) MII oocyte counts. Similar outcomes were obtained when analyzing gonadotropin dosing either as a cumulative dose or considering the starting and continuation doses separately.

Consistent with our findings, previous studies have shown a negative correlation between the total gonadotropin dose used in assisted reproductive technology and the number of retrieved oocytes [21]. Patients receiving 1001–2000 IU of total gonadotropin during ovarian stimulation yielded the highest number of retrieved oocytes, whereas doses exceeding 5000 IU significantly reduced this outcome by as much as half. Our results showed that patients with low (1–3 oocytes) and intermediate (4–8 oocytes) MII prediction did not benefit from increasing gonadotropin doses. Interestingly, patients with the highest MII predictions (9–12 oocytes) achieved better outcomes with a mid-range cumulative gonadotropin dose compared with the lowest 1050 IU and highest 2100 IU doses.

The subgroup of patients with the lowest MII prediction is of particular concern; these patients often receive high gonadotropin doses to stimulate follicle development. However, our findings challenge the assumption that maximizing the dose is as effective as generally believed, which aligns with previous studies showing that increased gonadotropin doses did not enhance the ovarian response in predicted poor responders [29, 30].

The gonadotropin dosing regimen for patients with high MII oocyte prognosis should also be carefully considered, as this subgroup is at a heightened risk of ovarian hyperstimulation syndrome. In our study, the most beneficial effect on the retrieved MII oocyte count obtained in this group was observed when a daily FSH dose of 225 or 300 IU was administered. These patients benefited more when the total dose of gonadotropins administered during stimulation was in the range of 1275–1800 IU, suggesting that, in patients with a high predicted MII, the choice of a continuation dose of gonadotropin may be of greater importance. As noted in [21], about 80% of patients in their study received FSH doses outside the optimal range, raising significant concerns regarding patient safety. Furthermore, several studies have shown that increasing gonadotropin dosage negatively affects clinical pregnancy rates, live birth rates [31, 32], and the production of fertilized or good-quality embryos [31, 33]. Recently, the shift towards milder stimulation approaches has been widely discussed [34]. Stimulation with lower FSH doses (⩽150 IU/day) has been shown to offer similar IVF success rates compared to conventional high-dose regimens, but with a reduced risk of ovarian hyperstimulation [35]. Our findings align with this emerging trend, highlighting the diminishing role of high-dose gonadotropin treatments and the growing importance of milder protocols.

Comparisons of natural and high-dose gonadotropin-stimulated IVF cycles in humans demonstrated that gonadotropin stimulation can influence oocyte maturity, fertilization, and cleavage-stage embryo morphology [36]. However, the authors did not differentiate between FSH doses separately. Elevated daily gonadotropin doses are associated with a higher proportion of immature oocytes [37].

The lack of increased MII oocyte retrieval with higher doses of FSH during ovarian stimulation can be attributed to various factors. Individual responses to gonadotropins can greatly differ due to follicular sensitivity, which can be influenced by genetic variations in genes encoding hormone receptors, such as FSHR and *LHCGR*. Certain sequence variants in these genes have been linked to altered ovarian responses, affecting retrieved oocyte counts, stimulation duration, and FSH consumption [38]. Specific genotypes were associated with an increased ratio of FSH dosage to the collected oocyte count [39], whereas others might benefit from increased doses [40, 41]. To better understand these factors’ influence on patient responses and their impact on gonadotropin dosing efficacy, further research is warranted.

An additional aspect revealed by our study is the use of a machine learning tool to predict the number of mature oocytes, serving as an aid in decision-making regarding gonadotropin dosage. Our research uncovers another important aspect of optimizing ovarian stimulation: using a machine learning tool to estimate the number of mature oocytes as a factor in determining the prescribed dosage of gonadotropins. We demonstrated that an ML tool can accurately predict the number of MII oocytes retrieved after stimulation. The most important features for this prediction are AMH and AFC, which are commonly used to determine gonadotropin doses [42, 43]. However, by including less influential features, we captured their cumulative effects on prediction. As many of these features are interrelated, incorporating them into the model enhances its predictive accuracy.

Utilizing ML tools to predict oocyte numbers presents several advantages for guiding gonadotropin dosing strategies in IVF, thus enabling personalized treatment plans. Analyzing multiple variables, such as patient characteristics, biomarkers, and historical data, has the potential to lead to more informed clinical decisions and reduce trial-and-error approaches. ML models can help optimize gonadotropin dosages to obtain maximum efficacy while minimizing risks, such as OHSS, potentially improving resource use, and lowering treatment costs. However, the clinical integration of ML tools will require rigorous validation, continuous monitoring, and multidisciplinary collaboration between data scientists, clinicians, and reproductive health experts to ensure the reliability of these predictive models and patient safety.

The strength of this study lies in the utilization of our previously developed machine-learning model, which provides highly accurate predictions of the number of MII oocytes [25]. The choice to utilize the ML model was driven by its ability to capture complex nonlinear relationships between multiple clinical factors, such as age, BMI, previous stimulation outcomes, and conditions, such as endometriosis, PCOS, and other causes of infertility. These factors collectively enhance the accuracy of MII oocyte predictions, surpassing the capabilities of traditional statistical methods, particularly in handling the intricacies of our large and complex dataset. Furthermore, by using predictions from our machine learning model to create study groups, we ensured that patients within the same group had highly similar characteristics encompassing various fertility-related features. This approach allowed us to establish a direct relationship between patient prognosis, gonadotropin dosage, and stimulation outcome, as all other patient characteristics were already encompassed within the model-based prediction. Our study also supports the idea of using machine learning models to precisely predict stimulation outcomes for optimizing gonadotropin dosing selection, as recently reported [31]. These authors demonstrated that employing the machine learning model for dose selection resulted in higher numbers of mature oocytes, fertilized embryos, and usable blastocysts, while reducing the amount of starting and total FSH used.

A limitation of the method presented in our study is related to model bias. Regression models tend to be biased towards the mean value in the studied population, so the trained model is inclined to generate lower predictions for patients with the highest expected MII and inflate the number of MII predicted for patients with the lowest expectations. Additionally, gynecologists are more inclined to assign the highest doses to patients with low MII oocyte retrieval expectancy; therefore, the size of the group of patients with a predicted MII oocyte count < 4 who received a dose of 300 IU/day was significantly larger than that of the group receiving lower doses administered under the same MII predictions. Another limitation is that gynecologists who prescribe higher doses may possess additional knowledge that was not recorded in the data. Our data should be interpreted with caution because our analysis was limited to patients with MII oocyte predictions of up to 12 oocytes. The use of higher doses of gonadotropins may lead to ovarian hyperstimulation syndrome [44], which is particularly possible in patients with higher predicted MII counts. Therefore, further studies should include such patients to determine whether our observations apply to those with higher MII prognoses. Additionally, to avoid bias, our study excluded stimulations with extreme AMH or AFC values. This limits the generalizability of our model to outliers that may occur in some patients. Therefore, a dedicated model and analysis of such cases are required.

A potential limitation of our study was the heterogeneity of the study population, arising from the division of stimulations into nine distinct gonadotropin-dosing regimens. Some dosing regimen groups had relatively small numbers of participants. To mitigate potential biases, we excluded groups with fewer than 30 individuals from certain analyses and separately examined the effects of starting and continuation FSH doses in the larger groups. The inclusion of diverse protocols reduced the risk of selection bias, thereby ensuring that the study captured a wide range of patient responses. The diversity in stimulation protocols created a rich dataset for exploring dose–response relationships, allowing for a nuanced analysis of how varying gonadotropin dosages impact ovarian stimulation outcomes and provide a more comprehensive understanding of the treatment effect. Moreover, this study was based on a dataset obtained in a real-world clinical setting where diverse conditions are commonly encountered. To verify our findings, we suggest that a prospective study be performed where a prediction is first performed and then different doses of gonadotropins are administered to patients with the same prediction. It would also be worth determining how such doses affect other IVF success outcomes, such as the number of high-quality embryos obtained or live birth rate.

Another limitation is the distinctive characteristics of the study population, which is marked by a relatively high prevalence of genetic defects and endocrine disorders. This may constrain the generalizability of our findings to populations with different prevalence rates of these characteristics. However, our primary focus was to evaluate the impact of gonadotropin dosing on ovarian stimulation outcomes, with genetic factors considered integral to the overall patient profile. Thus, these factors were included as covariates in the analysis to account for their potential influence on the stimulation outcomes. Further research is important to study diverse populations to reflect real-world clinical scenarios in which patients may present with various genetic and endocrine conditions.

In conclusion, our results show that increasing gonadotropin doses for ovarian stimulation did not enhance the efficiency of MII oocyte retrieval beyond the predicted number. In the future, the application of ML models may enhance gonadotropin dosing precision, thereby avoiding unnecessary increases in medication and improving treatment cost efficiency.

## Data Availability

INVICTA Fertility Clinics do not allow public disclosure of patient data used in this study. In case of additional questions, please contact the authors or INVICTA Research and Development Center (cbr@invicta.pl).
